# From fitness to fate: Hippo-YAP-mediated cell competition influence on stem cells activity

**DOI:** 10.1042/BSR20253960

**Published:** 2026-01-28

**Authors:** Apapist Panichewa, Chanchao Lorthongpanich

**Affiliations:** 1 Siriraj Center of Excellence for Stem Cell Research, Department of Medicine, Faculty of Medicine Siriraj Hospital, Mahidol University, Bangkok, 10700, Thailand; 2 Blood Products and Cellular Immunotherapy Research Group, Faculty of Medicine Siriraj Hospital, Mahidol University, Bangkok, 10700, Thailand

**Keywords:** apoptosis, cell competition, cellular fitness, Hippo pathway, YAP

## Abstract

Cell competition is a fundamental quality-control mechanism where fitter ‘winner’ cells eliminate less-fit ‘loser’ counterparts, thereby fine-tuning cell populations during development and maintaining adult tissue integrity. This highly conserved, natural cellular process is absolutely crucial for organogenesis, but once dysfunctional, can instead be exploited by ‘super-fit’ cancer cells to promote tumourigenesis. This review aims to provide an overview of how heterogeneity is the root cause of cell competition, the factors which influence its emergence, the various modes of cell competition, and finally, the mechanisms by which loser cells are eliminated. We are particularly interested in YAP, a major effector of the Hippo signalling cascade, as a driver of heterogeneity and perpetrator of human pluripotent stem cell (hPSCs) competition. We discuss how differential YAP/TEAD activity, influenced by mechanical stress, defines winner and loser cell identities within stem cell populations. Finally, we discuss the potential of cell competition for advancing regenerative medicine and cancer therapy.

## Introduction

Tissue development and maintenance is influenced by various intrinsic and extrinsic factors. It has become increasingly evident that cellular interactions within heterogenous tissue greatly shape its composition and function. Among these interactions, cell competition has emerged as a fundamental quality control mechanism whereby cells compare their relative fitness, leading to the elimination of less-fit or loser cells by more-fit winner counterparts [1–4]. This process drives early development and ensures tissue integrity in adult organisms [1,4–6].

**Figure 1:**
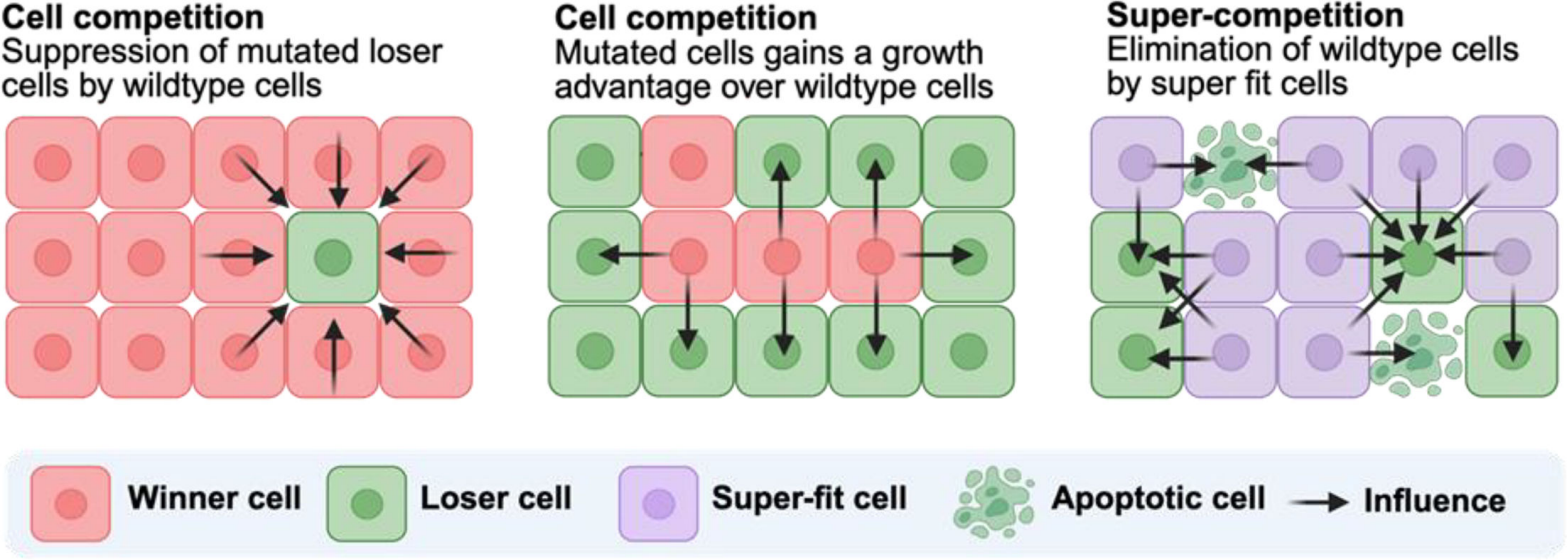
Winner and loser cell behaviors in cell competition. Cell competition enables 'winner' cells to outcompete and eliminate neighbouring 'loser' cells, contributing to either tissue homeostasis (cell competition) or, conversely, to cancer progression (super-competition model).

This phenomenon was first described in *Drosophila melanogaster* by Morata and Ripoll in 1975, where they observed competitive behaviour between wildtype cells and those lacking crucial ribosomal *Minute* gene [7]. Around the early 2000s, cell–cell contact-dependent competition was gaining traction. Starting with an investigation by Johnston in 1999, where he revisited *Minute* mutation in drosophila and found that loser-cell elimination occurs via apoptosis, and importantly, only at the clone boundary, suggesting an interaction-based process [8]. The conservation of this mechanism in mammals was later confirmed by Oliver’s group, who identified a Minute-equivalent ribosomal defect in mouse cells that similarly conferred a loser phenotype [9]. Notably, in disease states, particularly cancer, this behaviour has been found to be exploited by genetically unstable, highly proliferative, ‘super-fit’ cancer cells to eliminate surrounding healthy diploid cells (Figure 1), thereby facilitating tumour growth and metastasis [1,5,10–12].

Initially discovered in model organisms [7], cell competition is now recognized to play essential roles in developmental biology, stem cell regulation [13–16], lineage decisions [6,10], and the progression of diseases particularly in cancer [17–19]. The outcome of cell competition is not only restricted to cell survival versus cell death; instead, it is also important to consider how loser cells may be eliminated through the switch between self-renewal, quiescence and differentiation states [2,20–22]. As the field continues to expand, understanding the molecular and mechanical web behind cell competition holds significant promise for advancing regenerative medicine and cancer therapy. Among the molecular regulators of cell competition, the Hippo–YAP pathway has emerged as a unique integrator of mechanical, metabolic and density-dependent cues, positioning it as a central determinant of winner–loser identity across tissues.

In this review, we provide an overview of the concept of cell competition, including how it emerges, the types of cell competition that occur and the mechanism by which loser cells are eliminated. We are especially interested in its influence on cell fate decisions in various stem cell populations, including human pluripotent stem cells (hPSCs). Finally, we explore the emerging involvement of the Hippo signalling pathway in mediating cell competition, given its central function in contact inhibition and mechanosensing regulators [23–26].

## Cell competition: the mechanisms underlying cellular fitness

**Figure 2:**
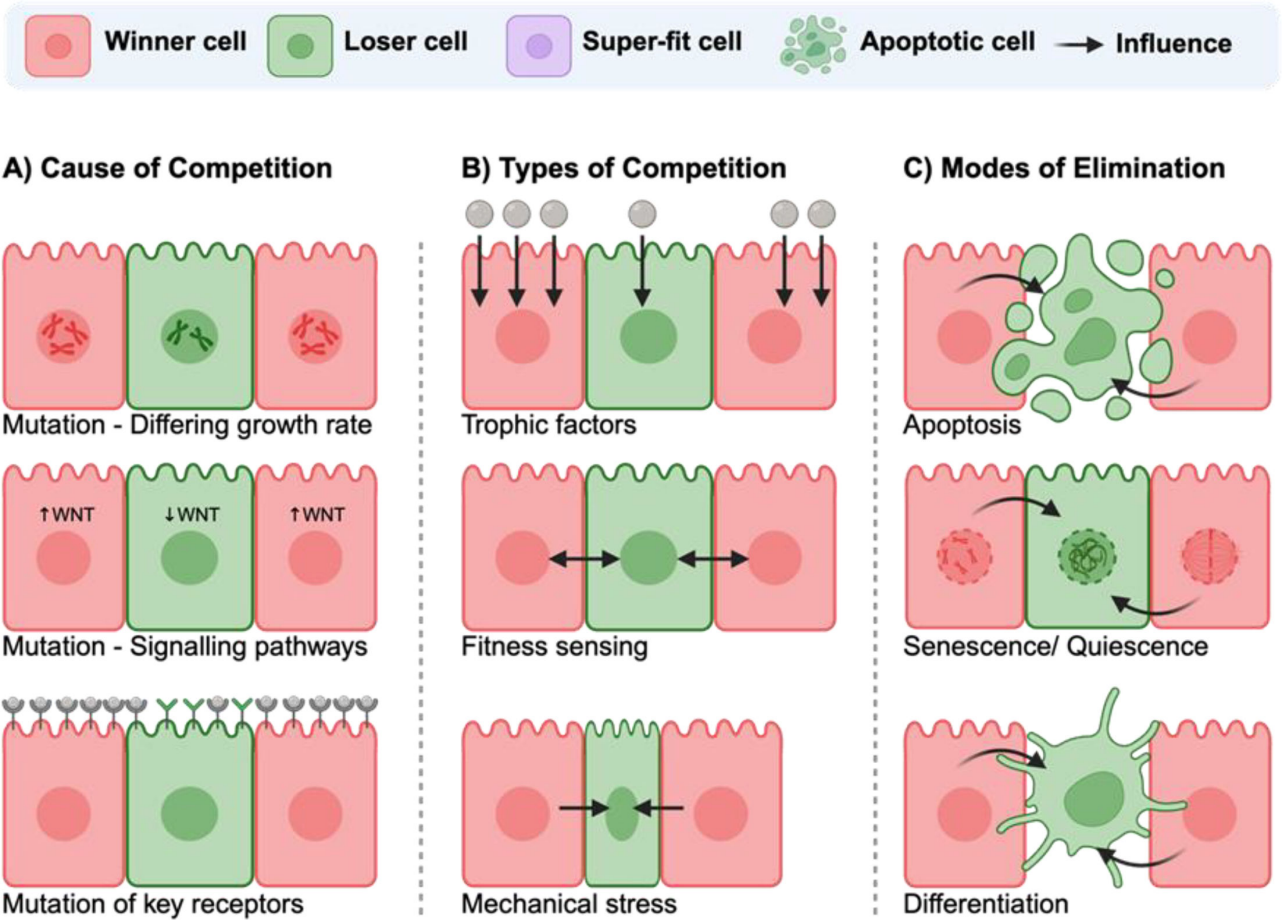
The three main types of cell competition and modes to loser-cell elimination. (**A**) Winner (red) and loser (green) cells can compete through various methods; trophic factors competition, (**B**) direct sensing each other level of fitness level or mechanically compression. As a result, loser cells are eliminated through apoptosis (**C**), enter a senescence or quiescence programme or are become differentiated.

Winner cells outcompete and eliminate neighbouring less fit or ‘loser’ cells through three main modes of cell competition: competition for trophic factors, direct fitness-sensing competition and contact-dependent or mechanical competition [1,4,5], detailed in (Figure 2). This mechanism plays a crucial role in organogenesis, by ensuring that only the healthiest cells contribute to tissue formation and maintenance, while damaged, mutated or oncogenic cells are removed [1,4,5]. The defining characteristics and molecular bases of each of these three models are described in the following sections, with particular emphasis on how mechanical competition provides a mechanistic bridge to Hippo–YAP signalling.

Cell competition can be conceptualized as arising from either cell-autonomous or cell–cell interaction-based mechanisms (Figure 2). Cell-autonomous competition is driven by intrinsic cellular properties such as genetic integrity, proteostasis, metabolic state or baseline signalling activity, which predefine a cell’s competitive fitness. Examples include ribosomal defects, proteotoxic stress or oncogenic signalling imbalances that render cells intrinsically less fit.

In contrast, interaction-based competition depends on relative fitness and emerges through direct interactions with neighbouring cells, including competition for trophic factors, molecular fitness-sensing systems and mechanical cues such as crowding, corralling and compressive stress (Figure 2A–C). Importantly, these modes frequently co-operate, as intrinsic fitness differences are amplified through cell–cell interactions to generate winner–loser outcomes. Notably, mechanical competition and Hippo–YAP signalling integrate autonomous and non-autonomous cues, translating intrinsic fitness states and extrinsic mechanical forces into context-dependent cell fate decisions.

### Competition for trophic factors

Trophic factor-mediated cell competition is one of the earliest recognized modes of cell competition in which cells compete for limited nutrients, cytokines or growth factors within their microenvironment (Figure 2A). When these resources are scarce, less competitive or fewer fit cells are deprived and undergo programmed cell death. A model was first described by Raff in 1992, who suggested that the number of neurons is regulated by access to neuronal survival factors such as nerve growth factor (NGF), brain-derived neurotrophic factor (BDNF), or ciliary neurotrophic factor (CNTF). This competition triggers programmed cell death, thereby limiting neuronal populations to levels an organism can sustain [27]. According to this model, developing neurons that fail to access sufficient trophic support undergo apoptosis, thereby ensuring that the number of surviving neurons is matched to the target field’s capacity to sustain them.

More recently, trophic competition has been identified as a tumour suppressive mechanism in the immune system. In the thymus, newly generated T cells emerging from thymic selection compete for the cytokine interleukin-7 (IL-7), a critical factor for T cell survival and homeostasis. IL-7 is limited and presented by IL-7-expressing stromal cells, including thymic epithelial cells. Newly differentiated T cells, which typically express higher levels of IL-7 receptor (IL-7Rα), outcompete older resident T cells for IL-7, leading to apoptosis of the latter [28]. This ensures a dynamic turnover of the T cell repertoire and prevents clonal overexpansion, doubling as a tumour suppressive mechanism in the thymus. Dysregulation of trophic factor competition has also been implicated in disease. For instance, aberrant IL-7 signalling has been associated with T-cell acute lymphoblastic leukaemia (T-ALL), where malignant cells hijack this survival pathway to gain a competitive advantage over normal T cells. Collectively, these examples demonstrate that competition for trophic factors is a critical and evolutionarily conserved mechanism by which tissues regulate cell number, maintain cellular diversity and prevent malignant transformation through selective survival cues. While trophic factor competition relies primarily on extracellular resource limitation, it lacks an intrinsic mechanism for translating physical cell–cell interactions into transcriptional fitness programmes, distinguishing it from mechanically driven modes of competition.

### Direct fitness-sensing competition

Direct fitness-sensing competition is a passive form of cell competition in which ‘winner’ cells detect and respond to the diminished fitness of neighbouring ‘loser’ cells, often resulting in their apoptotic elimination (Figure 2A,B). This process is mediated by specific molecular signals that allow cells to compare their relative fitness and selectively eliminate suboptimal neighbours.

This mechanism was first characterized in *Drosophila* imaginal wing discs through an unbiased genetic screen, which identified a transmembrane protein called Flower as a key mediator of this process. Different isoforms of Flower mark cells as either winners or losers, enabling healthy cells to recognize and eliminate their less-fit neighbours [29]. This discovery elucidated the molecular basis of Minute-mediated cell competition, wherein wildtype cells eliminate *Minute* ^+/-^ cells, in which the defective ribosomal protein genes were observed, due to their impaired protein synthesis and slower growth rates [1,29–31]. Following the identification of Flower, additional fitness-sensing proteins have been discovered. One such protein is Azot (*ahuizotl),* a cytoplasmic protein in *Drosophila* that functions downstream of Flower to execute apoptosis in loser cells. Azot is up-regulated in looser cells especially during aging and is necessary for their elimination. This phenomenon further supports the idea that cells actively monitor their neighbours’ fitness and trigger a defined elimination programme [32]. Sparc (Secreted Protein, Acidic and Rich in Cysteine) is a secreted protein that interacts with the extracellular matrix (ECM), influencing how cells interact with their environment. It has also been implicated in modulating cell competition in certain contexts by influencing extracellular matrix composition and cell survival [33]. This crucial piece of the puzzle not only adds a more complete picture of classic studies of this field [7–9], it also points towards the need for understanding cellular components as it can detect growth differentials and causes cell competition, which then affects, at the macro level, the overall tissue development and organization. Importantly, fitness-sensing mechanisms have been observed in mammalian systems. Sancho et al. demonstrated that mouse embryonic stem cells (ESCs) with impaired BMP signalling were eliminated when co-cultured with wildtype ESCs, through a mechanism involving fitness comparison and apoptosis induction [15]. This suggests that mammalian cells, like their *Drosophila* counterparts, can engage in molecular-level comparisons to preserve tissue quality. Thus, direct fitness-sensing competition represents a highly conserved surveillance mechanism that not only regulates developmental processes but may also underlie pathological cell selection in cancer and degenerative conditions. Although direct fitness-sensing competition relies on molecular recognition systems, such as Flower and Azot, it often converges with mechanical and cytoskeletal cues that ultimately influence transcriptional regulators of cell fate.

### Contact-dependent or mechanical competition

Contact-dependent or mechanical competition arises from defined physical forces rather than a generic mechanical state, distinguishing it from competition for soluble factors or molecular fitness markers alone. Specific mechanical determinants contributing to loser cell elimination include spatial confinement and crowding, differential proliferative pressure, cell corralling and compaction, cytoskeletal tension, substrate stiffness and compressive or shear stress imposed by neighbouring cells (Figure 2B). These forces act individually or in combination to reduce cellular volume, disrupt cytoskeletal organization and impair mechanotransductive signalling, thereby biasing affected cells toward extrusion, growth arrest or apoptosis (Figure 2C).

In mosaic tissues or densely packed epithelia, faster-growing or mechanically advantaged winner cells generate increased solid stress that physically compresses neighbouring loser cells, leading to cell shrinkage, extrusion or apoptosis (Figure 2C) [3,19,31,34,35]. These mechanical stresses are sensed through cytoskeletal remodelling and actomyosin contractility, which alter intracellular tension and activate mechanotransduction pathways.

Importantly, mechanical competition is governed by reciprocal feedback between mechanical forces and intracellular signalling, most notably the Hippo–YAP axis. Cell stretching, reduced confinement and increased substrate stiffness promote nuclear YAP localization and transcriptional activity, enhancing cellular fitness, whereas compression and corralling drive YAP cytoplasmic sequestration and loss of proliferative capacity in loser cells (Figure 2B). In turn, YAP signalling feeds back to regulate cytoskeletal organization, intracellular pressure and cell growth, thereby reinforcing mechanical asymmetries between winner and loser cells. This bidirectional signalling–mechanics feedback loop provides a mechanistic framework through which physical forces are translated into transcriptional programmes that define competitive cell fate decisions.

The concept of mechanical competition was first proposed by Shraiman [36], who predicted through computational modelling that, in space-limited tissues, faster-growing cells would outcompete and induce apoptosis in slower-growing neighbours, resulting in uniform tissue growth rates [1,12,34]. This model has since been experimentally validated across multiple systems. In epithelial tissues, cell crowding promotes extrusion and elimination of less-fit cells from the epithelial sheet [31,37].

Similarly, in immortalized mammalian cell lines, subclones with higher proliferative capacity generate mechanical stress that leads to the selective elimination of loser clones [38]. More recently, Zhu and colleagues suggested that innate immune cells can contribute to epithelial cell competition by selectively targeting and eliminating less-fit cells, extending classical mechanical competition concepts to immune-mediated surveillance contexts [37].

Together, these findings indicate that mechanical cell competition should be viewed as a spectrum of context-dependent biophysical selection pressures, rather than a single generic mechanism. Although trophic, molecular fitness-sensing and mechanical competition differ in their initiating cues, they converge on a shared principle of relative cellular fitness. Mechanical competition is unique in that it directly interfaces with Hippo–YAP signalling, providing a mechanistic framework through which physical forces are converted into stable winner–loser cell fate decisions.

## Factors influencing competition

Heterogeneity within a dish, tissue or organ system is often the root cause of cell competition, generating relative fitness differences that precede and drive competitive interactions [2]. This heterogeneity arises from a range of intrinsic and extrinsic stimuli that influence the relative fitness of individual cells (Figure 2A).

### Genetic mutations and cellular stress

Mutations can profoundly affect a cell’s ability to survive and proliferate, often sparking cell competition by introducing heterogeneity within a population. Taking an earlier mentioned example of *Minute* in the drosophila, the loser population possesses a heterozygous *Minute^-/+^* mutation resulting in lower proliferative capacity allowing elimination by winner wildtype *Minute^+/+^* cells [7]. Initially, this loser phenotype was attributed to decreased ribosomal translation. However, later studies revealed that the true driver was increased proteotoxic stress within the mutant cells [39]. This illustrates how genetic mutations can reduce cellular fitness, thereby triggering their elimination through cell competition. Similar phenomena were observed in mammalian systems. Common karyotypic abnormalities in human pluripotent stem cell (hPSCs) cultures, such as gain of chromosome 1, 12, 17, 20 and X, have been shown to confer a growth advantage to affected cells [14,40]. These chromosomal abnormalities often generate a fitness disparity, allowing the genetic mutated cells to out-complete their diploid neighbours.

### Differential signalling pathway activation

Disparities in the activation of key signalling pathways can also drive cell competition. For instance, differential WNT activity between adjacent cells of the *Drosophila* wing disc can initiate competitive interactions. Cells with reduced WNT pathway activation become at a disadvantage compared with their neighbours with normal or high WNT signalling. These loser WNT-low cells are eliminated via apoptosis by neighbouring normal/high WNT cells [27]. Another prominent signalling axis involved in cell competition is the Hippo pathway, due to its control of organ size, cell proliferation and apoptosis. The Hippo signalling pathway plays a crucial role in cell competition through its downstream effectors YAP (Yes-associated protein) and TEAD (TEA domain transcription factors). When the Hippo pathway is inactivated (Hippo off), nuclear accumulation of YAP/TEAD promotes the expression of anti-apoptosis and survival genes, enhancing cellular fitness and giving rise to a super-competitor phenotype. These hyperactive cells not only gain a proliferative advantage but also actively eliminate neighbouring less-fit cells. Conversely, mouse fibroblast cells with reduced TEAD activity exhibit diminished proliferative capacity and are selectively removed by surrounding wildtype cells. This bidirectional behaviour underscores the Hippo-YAP/TEAD axis as a central fitness-sensing mechanism in cell competition [6,41]

In addition to the well-characterized WNT and Hippo pathways, several other signalling cascades have been implicated in cell competition. Myc, a potent transcription factor downstream of various growth pathways, can induce a super-competitor phenotype when overexpressed, enabling winner clones to aggressively outcompete and eliminate their wildtype neighbours in both *Drosophila* and mammalian systems [42–45]. BMP and TGF-β signalling, which govern growth and differentiation, may influence competition by altering the tissue microenvironment or modulating cell fate responsiveness [46]. Additionally, variations in Notch and EGFR pathway activity have been shown to affect cell adhesion and proliferation, contributing to competitive dynamics in certain developmental and *in vitro* contexts [47]. These findings underscore the complexity and versatility of signalling-based fitness cues that govern cell competition across diverse biological systems.

### Cell polarity and structural defects

Cell polarity is crucial for maintaining epithelial tissue integrity, regulating cell division orientation and ensuring proper localization of signalling molecules [26,48]. Defects in epithelial polarity and changes in cell architecture can trigger competitive behaviour. It has been shown that mutation of *Scribble* in *Drosophila* imaginal wing disc, which causes a loss of apico-basal polarity, leads to the displacement of TNF receptor Grindelwald, making the mutant cells more susceptible to TNF ligand-induced apoptosis. The excessive ligand–receptor interaction activates the JNK signalling cascade leading to the elimination of the polarity-defective cells via wildtype neighbours [6,49]. Recently, Yamamoto and colleagues [50] reported the upstream mechanism regulating polarity loss triggering the apoptosis via the competitive interaction at the boundary of the cells. Their study identifies the Sas–PTP10D ligand–receptor system as a key polarity-sensitive mechanism. They found that Sas localized in winner cells and PTP10D localized in loser cells, enabling their interaction at the lateral membrane. This interaction suppresses EGFR signalling in polarity-deficient cells, allowing JNK signalling to promote their elimination. Without this polarity-dependent signalling, JNK instead drives tumour-like overgrowth. Thus, loss of polarity marks cells as less fit, and cell competition eliminates them through a mechanism requiring intact polarity cues in neighbouring cells. The elimination of polarity-defective cells through JNK signalling is not merely a response to cell-autonomous defects but often requires the presence of neighbouring ‘winner’ cells, emphasizing the relative nature of cell competition. This suggests that epithelial tissues constantly compare the polarity and structural integrity of adjacent cells to preserve tissue homeostasis. In certain contexts, mislocalization of apical–basal polarity does not always lead to the elimination of affected cells as losers. Instead, in epithelial tissues, disruption of cell polarity components, such as PAR, Crumbs (CRB), Scribble (SCRIB) and aPKC can confer a growth advantage, enabling these cells to behave as super-competitors and contribute to tumourigenesis [28,51,52]. Together, these findings highlight the dual role of cell polarity disruptions in cell competition either acting as a cue for the elimination of less fit cells under normal conditions or promoting competitive advantage and tumour progression under certain oncogenic contexts, thereby underscoring the context-dependent outcomes of polarity loss in epithelial tissues. Collectively, these findings underscore that cell competition is fundamentally context-dependent, with polarity loss functioning either as a loser signal or as a driver of super-competitor behaviour depending on tissue state and oncogenic background.

## Cell competition shapes stem cell fate decisions beyond apoptosis

Beyond direct elimination by apoptosis, cell competition can also bias cells toward alternative fate decisions, particularly in stem cell systems where self-renewal and differentiation represent intrinsically unstable states.

Stem cell systems represent a unique context for cell competition, as competitive interactions do not necessarily result in immediate apoptosis but can instead bias self-renewal, differentiation, quiescence or senescence. In many studies, the dominant mode of competition is activation of apoptotic pathways in loser cells [14,53,54].

However, here we discuss the potential effects of cell competition on the balance between self-renewal and differentiation within stem cell populations (Figure 2C) [2,20–22]. Recently, Khandekar and Ellis [2] proposed that cell competition should not be viewed merely as a binary process in which winner cells eliminate losers via apoptosis. Instead, they describe a spectrum of competitive interactions, where tissues exhibit a gradient of cellular fitness [2]. Pushing the idea that subtle differences in gene expression, metabolism and epigenetic regulation drive competitive dynamics without overt cell elimination [1,2,6]. According to this model, transcriptional stochastic variability, including enhancer activity and epigenetic interactions, establishes heterogeneity within stem cell populations [30,55], influencing relative cellular fitness and ultimately fate decisions.

Supporting this view, Hu and colleagues [55] demonstrated that the transcription factors Klf4 and Zfp281 act antagonistically at differentially active transcribed enhancers (DATEs), regulating gene expression variability in mouse embryonic stem cells. This enhancer-mediated antagonism shapes the dynamic interconversion between naïve and primed pluripotent states, suggesting that heterogeneity in transcription factor activity can bias cells toward different self-renewal or differentiation trajectories, thereby contributing to fitness differences that may be acted upon through cell competition.

Although pluripotency is inherently unstable cell state [56–59], early mammalian embryos manage to keep their pluripotent state through this MYC-mediated cell competition, eliminating any cells that are poised for differentiation [13,41,42,60]. Myc levels are correlated with pluripotency and the differences in Myc expression can trigger competitive interactions between naïve (Myc-high) and primed (Myc-low) cells [13]. Persistent cell–cell contact between naïve and primed populations induces apoptosis in MYC-low primed cells, further supporting MYC’s role as a regulator of both human and embryonic stem cell competition [13].

A similar paradigm was observed in the bone marrow niche, where haematopoietic stem and progenitor cells (HSPCs) compete for access to membrane-bound stem cell factor (SCF), a key regulator of stem cell maintenance [61]. SCF is presented on the surface of niche-supporting cells such as mesenchymal stromal cells (MSCs) and endothelial cells, and it binds to the c-Kit receptor (CD117) on HSPCs [61,62]. This interaction is crucial for maintaining HSPC quiescence, proliferation and self-renewal.

Under conditions of limited SCF availability, only the most responsive or receptor-rich HSPCs persist, whereas others undergo apoptosis or differentiation. Suppression of loser HSPCs may also occur through induced senescence or differentiation (Figure 1C). In haematopoietic systems, less-fit HSPCs can be eliminated by complete withdrawal from the cell cycle and entry into a senescence-like state [10]. This competition serves to preserve the most functional stem cell clones and maintain haematopoietic homeostasis [61].

On the other hand, cancer expansion can involve forced differentiation of its wildtype counterparts [6,63,64]. For example, metastatic intestinal cancer cells induce differentiation of wildtype liver progenitors through compaction and cell-cycle arrest. These newly differentiated liver cells exhibit reduced cellular fitness and are therefore more prone to elimination, allowing cancer cells to outcompete the surrounding tissue [18].

## Mechanosensitive Hippo-YAP signalling as a central integrator of cell competition

Among the pathways translating fitness differences into competitive outcomes, the Hippo–YAP axis has emerged as a central mechanosensitive regulator that converts physical forces, cell density and cytoskeletal tension into transcriptional cell-fate decisions. Mechanical competition often results in cell shrinking and stretching, remodelling the cell’s cytoskeletal structure [19,34,36]. Cell stretching has been associated with increased growth and proliferation [23,65] while corralling impairs cell health, potentially inducing apoptosis from the shear stress [14,19,66,67]. These opposing mechanical inputs have been linked to the up-regulation [2,23,24,68] and down-regulation [14,69] of YAP activity respectively. These mechanical cues drive dynamic shifts in YAP localization, influencing tissue heterogeneity and cell fate decisions [14,23,70]. In combination with its effects on cytoskeletal tensions, YAP has also been reported to affect intracellular cytoplasmic pressure and therefore affect cell volume, which results in timely progression from G1 to S phase in the cell cycle [71]. Collectively, these findings raise the question of how central YAP, and the broader Hippo pathway, are in defining winner and loser cell identities during cell competition as well as how it influences the method of loser cell elimination.

**Figure 3:**
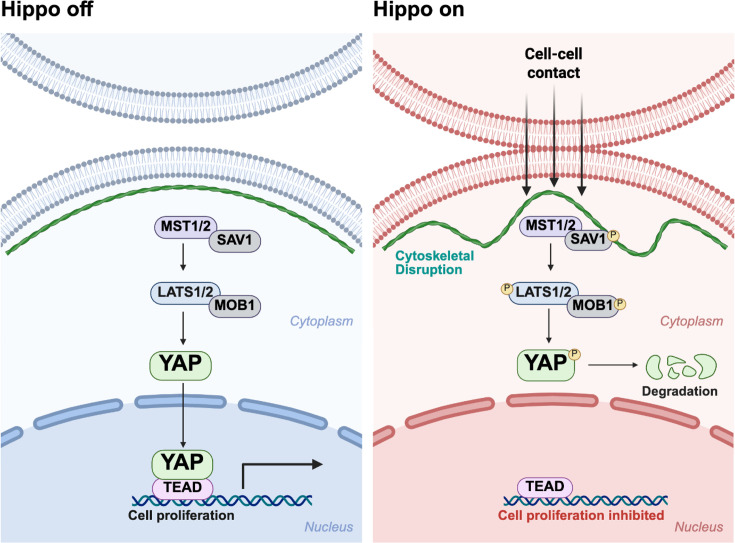
Hippo signalling cascade. When Hippo is off (blue), YAP translocates into the nucleus to form a complex with TEAD, transcribing cell proliferation genes. Once Hippo is switched on (red) from cell-cell contact or other mechanical cues, YAP is sequestered in the cytoplasm and degraded; therefore, cell proliferation is inhibited.

The Hippo pathway is a highly conserved kinase cascade responsible for cell proliferation, regeneration and organ size control across many species [47,72–74]. The disruption of Hippo signalling is therefore associated with various diseases including cancers, immune dysfunction, cardiac diseases, pulmonary diseases and renal diseases [47,75,76]. In mammals, the Hippo signalling cascade is activated through various upstream external signals (Figure 3), often in the form of mechanical or metabolic stress which then activates mammalian STE20-like kinase 1/2 (MST1/2) enabling it to form a complex with protein Salvador homologue 1 (SAV1) and activate large tumour suppressor kinase 1/2 (LATS1/2) MOBKL1A/B (MOB1A/B) complex. This then phosphorylates YAP or WW-domain-containing transcription regulator 1 (TAZ). In this instance (Hippo on), phosphorylated YAP is unable to translocate into the nucleus and therefore remain in the cytoplasm and gets degraded. When Hippo is turned off, YAP is able to enter the nucleus, binding to the transcriptional enhanced associated domain (TEAD) family1, resulting in transcription of YAP target genes, promoting cell survival and proliferation [24,47,74–77].

YAP functions as a potent transcriptional co-activator, regulating the proliferative programmes of tissue-specific stem cells [47,68,75,76] and oncogenesis [17,78]. Beyond its established role as a regulator of organ size, YAP regulates single cell size, volume and growth through non-cell autonomous mediators [71,79]. CYR61, a known YAP secreted molecule, effects cell proliferation and apoptosis at high density in HEK293 cells [79]. Its activity is highly responsive to mechanical stress imposed by neighbouring cells, as demonstrated across various model systems [14,23,78,80–85]. This mechanosensitive role of YAP was first described in mesenchymal stem cells (MSCs), where Dupont and colleagues demonstrated that extracellular matrix stiffness and cytoskeletal tension directly influence YAP localization, guiding MSC differentiation towards either adipogenic or osteogenic lineages [23]. Stiff substrate was reported to translocate YAP/TAZ into the nucleus [23,26,86], activating YAP activity and promoting MSC differentiation to osteoblasts [23,24,26,87,88]. On the contrary, soft substrates support YAP cytoplasmic retention and promote MSC to adipocyte lineage [23,24,26,87–90].

Furthermore, differential YAP localization has been linked to the naïve pluripotency stage in human embryonic stem cells [91]. In a recent study by [91], Arp2/3 complex remodels the actin cytoskeleton which allows YAP nucleus localization and transcription of hippo target genes, facilitating transition and maintenance of the naïve pluripotent stage. Once Arp2/3 is inhibited, the naïve pluripotent stage is impaired. However, these cells can be partially rescued through the forced expression of YAP as YAP restores the actin filaments and counters the effects of Arp2/3 inhibition [91]. These findings highlight the role of YAP in cytoskeletal remodelling and pluripotency maintenance. On the contrary, in the growing mouse epithelia, most basal epithelial cells harbour YAP cytoplasmic localization which biases the cells towards self-renewal rather than differentiation, driving proliferation [70]. Once the tissue size is finalized, the epithelium becomes increasingly compressed, inducing nucleus localization and shift towards differentiation [70]. Altogether, these observations suggest that YAP acts as a central integrator of mechanical cues in cell competition, where differences in cytoskeletal tension, substrate stiffness and spatial constraints influence YAP localization and, consequently, determine which cells thrive and which are eliminated.

## Hippo-mediated human pluripotent stem cell competition

**Figure 4:**
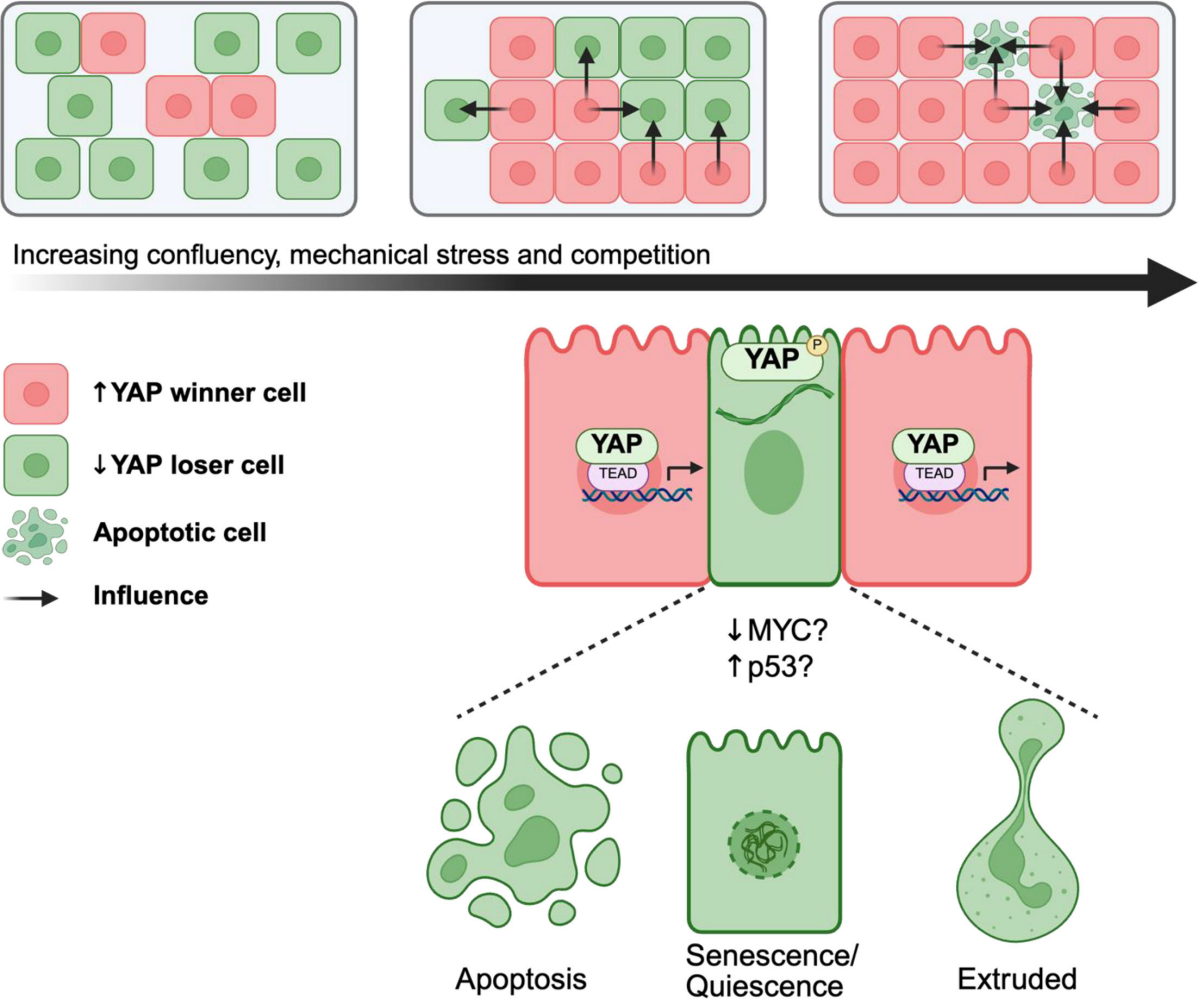
Cell competition model and Hippo YAP activity. As confluency increases in a dish, mechanical stress from neighbouring cells increases, triggering loser cell elimination. The relationship between MYC, p53 and YAP as facilitator of elimination remains to be explored. The method of elimination potentially includes apoptosis, senescence or quiescence and extrusion.

In *in vitro* hPSC cell competition, a study from Christopher Price’s group demonstrated that aneuploid winner clones conferred a growth advantage, while ‘loser’ cells underwent cytoskeletal redistribution, resulting in cytoplasmic sequestration of YAP and suppression of its transcriptional activity. Markedly, KEGG pathway analysis revealed that the Hippo signalling pathway was the only pathway significantly enriched when comparing differentially expressed genes between winner and loser cells in co-culture [14]. This reduction of YAP was found to facilitate the acquisition of ‘loser’ cell phenotype and subsequent elimination through apoptosis [14] (Figure 4). These findings support other observations that mechanical cue significantly modulates YAP localization and disruption of microtubules tends to promote cytoplasmic retention of YAP/TAZ [23,26,87,91].

Consistent with this, increased hPSC density reduces YAP and other pluripotency gene expression while enhancing differentiation-related programmes [87]. Furthermore, YAP depletion at high density reduces density-induced neural epithelial differentiation [87]. Interestingly, in the context of haematopoietic differentiation, YAP depletion enhances the differentiation efficiency and yield of haematopoietic stem and progenitor cells (HSPCs) in vitro [72].

In a parallel study, Mamada’s group established an in vitro model of Hippo-Myc-mediated cell competition using genetically manipulated mouse embryonic fibroblasts. Co-culture of high-TEAD (winner) and low-TEAD (loser) cell lines induced cell competition, marked by a local decrease in Myc expression and apoptosis in the loser compartment. They discovered that TEAD directly regulates Myc transcription, and that under low-density conditions, proliferation requires the activity of both TEAD and Myc. However, under high-density competitive conditions, suppression of either TEAD or Myc led to a compensatory up-regulation of the other. This compensatory mechanism was sufficient to restore competitive fitness and confer winner status to the cells [41]. Similarly, Price’s study demonstrated that promoting nuclear localization of YAP mitigated competitive elimination in hPSCs by increasing the resistance of potential loser cells to mechanical corralling and apoptosis. This highlights the central role of Hippo-YAP signalling in determining cell fate under competitive stress. Collectively, these findings underscore the pivotal role of Hippo-YAP signalling in mediating cell competition in pluripotent and somatic cells, where mechanical cues, cytoskeletal dynamics and transcriptional feedback orchestrate to determine cellular fitness, survival and lineage outcomes.

### YAP/TEAD, Myc and P53 in hPSC cell competition

**Figure 5:**
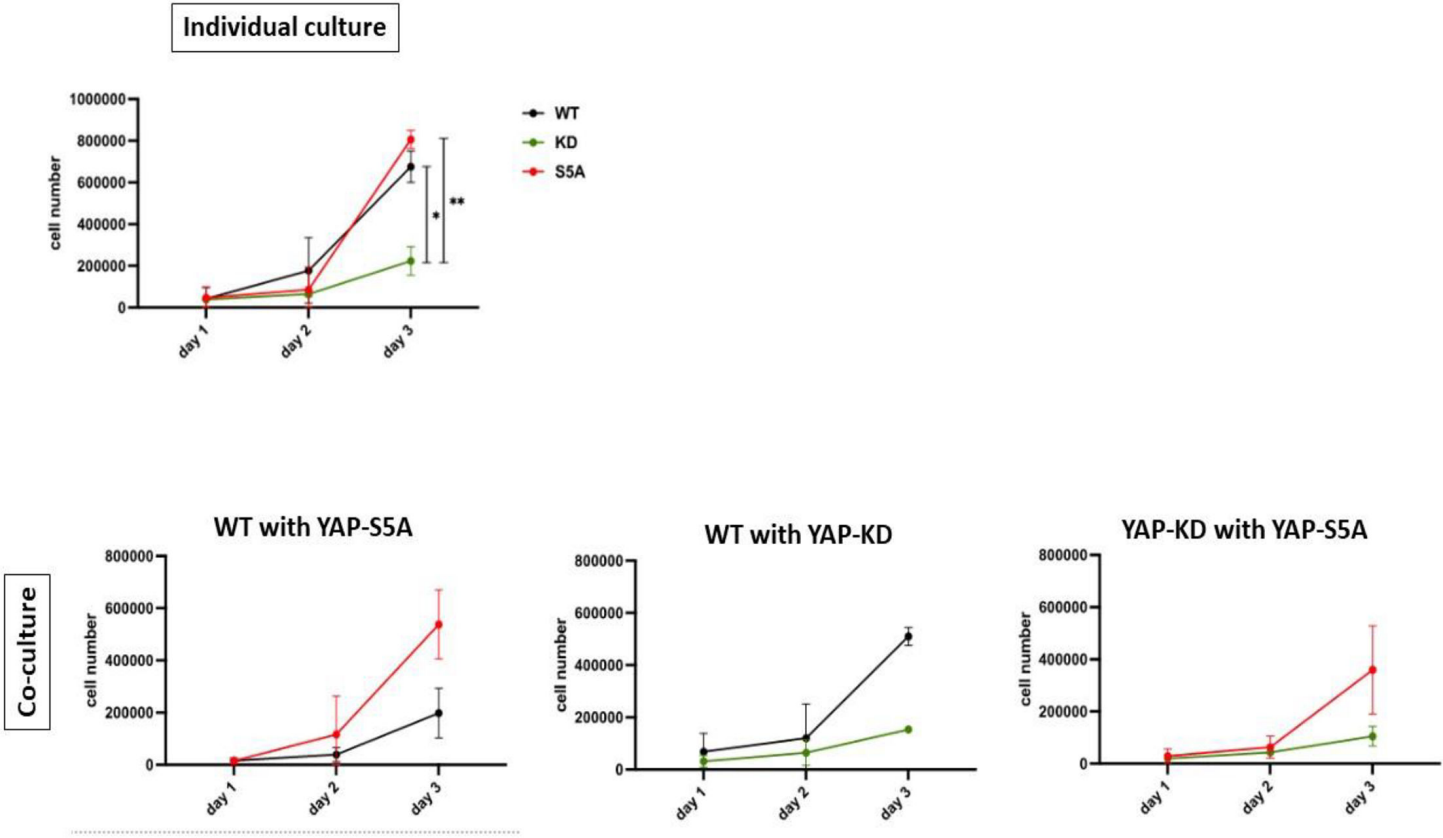
Reduced YAP expression drives a loser phenotype in high-density iPSC cultures. At d3 (high density), iPSCs with lower YAP expression has a lower proliferative capacity in monoculture and exhibited a ‘loser’ phenotype when co-cultured with cells expressing higher levels of YAP. Growth curve of wildtype (WT, black), YAP-knockdown (KD, green) and YAP-overexpressing (S5A, red) cells cultured separately and together. Cells were plated at 50,000 cells per well and the entire well was collected for flow cytometry at day 1, 2 and 3. Data represent mean ± standard error of mean from *n* = 2–3 independent experiments and analysed using two-way ANOVA, Sidak’s multiple comparison test. *P<0.05, **P<0.01, compared between cultures WT, KD and S5A conditions. d, day.

The role of aneuploidy in conferring a growth advantage during human pluripotent stem cell (hPSC) competition was further examined in a recent study by Ya et al. [16]. Unlike somatic cells, hPSCs can tolerate and proliferate despite aneuploid genetic profiles. Prolonged culture and serial passaging can lead to the emergence of winner clones harbouring specific chromosomal aneuploidies, which confer a selective growth advantage, allowing these clones to overtake their wildtype loser counterparts [14,16,40]. Consistent with these published findings, our own observations further illustrate how differential YAP expression can influence competitive outcomes in human iPSC cultures. Similar to Price’s findings, cells with higher YAP expression confer the winner phenotype and outcompete cells with lower levels of YAP, especially under confluent conditions (Figure 5).

In hPSCs, MYC is responsible for cell growth and maintaining pluripotency [13,60,92,93] whereas tumour suppressor protein, p53, plays a role in cell cycle arrest and recognizing DNA damage [10,12,16]. In early-passage mosaic cultures, non-autonomous cell competition between neighbouring diploid cells typically results in the elimination of low-MYC, high-p53 aneuploid cells [16]. However, with continued passaging, MYC expression can become up-regulated while p53 responses diminish, granting aneuploid cells a proliferative advantage and overtaking the entire culture [16]. The competitive elimination of cells with lower MYC levels by those with higher MYC expression has been well documented in multiple systems, including *Drosophila* wing discs [11,43], mouse embryos [42] and mouse embryonic stem cells [15].

Importantly, YAP/TEAD is an upstream regulator of MYC expression [41,83,94,95]. Over a decade ago, studies in *Drosophila* established a feedback loop wherein Yorkie (the YAP homolog) activates dMyc transcription to promote growth, while high levels of dMyc, in turn, repress Yorkie activity through genetic and epigenetic mechanisms [83]. In mammalian cells, YAP overexpression can partially rescue proliferation in YAP-deficient cells, highlighting MYC as a key effector, though insufficient to fully compensate for YAP loss [96]. Genome-wide binding analyses have shown that MYC and TEAD frequently co-occupy target loci, with YAP recruitment often dependent on MYC. Full transcriptional activation at these sites requires both factors [94]. This result underscores the co-operative nature of the YAP–TEAD–MYC axis.

Altogether, these findings suggest that heterogeneous YAP localization within a population enables the fine-tuned regulation of self-renewal, differentiation and apoptosis, which are critical for tissue development and homeostasis. This is especially relevant with Khandekar’s expanded definition of cell competition [2], which encompasses not only direct competitive elimination but also broader shifts in cell fate driven by fitness disparities. Dynamic changes in YAP signalling, amplified by non-autonomous cell competition, may serve as a mechanism to remodel tissue composition and ensure appropriate organ size. Overall, these studies reinforce the critical role of the Hippo-YAP/TEAD–MYC axis in mediating density-dependent, mechanical cell competition in stem cell systems.

## Hippo-YAP signalling in cancer cell competition

The oncogenic capacity of YAP is well recognized [75,78,97]. As the primary effector of the Hippo signalling pathway, YAP and its paralog TAZ are negatively regulated by the Hippo signalling pathway through phosphorylation by the core kinases MST1/2 and LATS1/2. This phosphorylation promotes their cytoplasmic sequestration or degradation, thereby preventing their nuclear accumulation and transcriptional activity (Figure 3). In oncogenic populations, disruption of Hippo pathway components, such as loss of NF2 or mutation of LATS1/2, can lead to constitutive activation of YAP/TAZ which enhances cellular proliferation and endows cells with a competitive advantage, making them super-fit [78,98]. For example, in epithelial cancers, disruptions in Hippo pathway components such as NF2, LATS1/2 or upstream regulators like FAT cadherins lead to constitutive YAP activation, tipping the balance toward pro-tumorigenic cell competition. Additionally, YAP can remodel the tumour microenvironment by altering immune cell recruitment and extracellular matrix composition, thereby creating a niche that further supports tumour progression [98]. A clear example of YAP-driven cell competition is seen in glioblastoma (an aggressive form of brain cancer), where xenograft models reveal that tumour cells with higher YAP expression eliminate neighbouring YAP-low cells through apoptosis. This results in clonal expansion of the more aggressive, YAP-high population and concurrent up-regulation of the oncogenic programme, contributing to tumour heterogeneity and adaptation [78].

Interestingly, YAP/TAZ are also implicated in tumour-suppressive competition under certain contexts. In metabolic dysfunction-associated steatotic liver disease (MASLD), a recent study demonstrated that YAP/TAZ activation in wildtype hepatocytes is triggered by metabolic and mechanical stress from surrounding tumour-initiating cells in mice models [17]. Rather than promoting transformation, YAP/TAZ activation can enhance cellular fitness and trigger competitive interactions that result in the apical extrusion and elimination of neighbouring oncogenic cells. Therefore, transient YAP activation may enable non-transformed cells to mount a protective response against malignant transformation. These findings highlight the dual functions of YAP/TAZ, which can either promote or suppress tumour growth depending on cellular context through a non-autonomous process. Overall, the role of Hippo-YAP signalling in defining winner and loser cell identities has broader implications in tissue homeostasis, stem cell renewal and cancer evolution.

## Therapeutic opportunities of cell competition in regenerative medicine and oncology

Cell competition has emerged as a promising therapeutic framework, offering opportunities to modulate cellular fitness, mechanical interactions and transcriptional programmes to improve tissue regeneration and suppress disease. Acting as a tissue quality-control mechanism, cell competition offers significant applications in regenerative medicine, where maintaining the integrity of stem cell populations is critical. Harnessing this mechanism could improve the safety and efficacy of stem cell transplantation by eliminating aneuploid or compromized cells, thereby enhancing tissue regeneration and accelerating wound healing. Promoting the dominance of healthy cells over senescent or damaged ones may also contribute to anti-aging strategies and improved recovery following injury.

Beyond these established roles, cell competition offers additional therapeutic opportunities. For instance, it could be exploited to selectively clear senescent cells that accumulate with age and drive chronic inflammation. Thus, serving as a novel senolytic strategy to rejuvenate tissues and ameliorate age-related diseases. In regenerative therapies, modulating cell competition to enhance stem cell engraftment by ensuring only genetically stable, highly fit cells persist and repopulate target tissues. Moreover, in fibrotic diseases, promoting the competitive elimination of pathogenic myofibroblasts could help resolve fibrosis in vital organs such as the liver, lungs and kidneys. Cell competition may also bolster immune defences by facilitating the removal of infected or virus-transformed cells through competitive interactions with healthy immune cells. Additionally, manipulating competition dynamics could suppress metastasis by favouring less invasive cellular phenotypes and help overcome cancer therapy resistance by preventing expansion of drug-resistant clones. Finally, understanding cell competition may enable the development of biomarkers to monitor tissue fitness and early malignant transformation, informing personalized therapeutic interventions.

In oncology, cell competition plays a dual role. During early tumourigenesis, it can act as a tumour-suppressive mechanism by identifying and eliminating potentially malignant cells. However, transformed cells may subvert this process and acquire ‘super-competitor’ status. This allows them to eliminate surrounding healthy cells and facilitate tumour progression. Future therapies might aim either to enhance this intrinsic tumour-suppressive surveillance or to reprogramme competitive interactions within tumours to render malignant cells less fit. This could be achieved by targeting key regulators such as Myc, YAP/TAZ or other metabolic pathways in conjunction with existing immuno-oncology therapies. While precise control of these context-dependent interactions remains challenging, targeting cell competition offers an innovative and multifaceted framework for cancer treatment, tissue engineering and management of age-related diseases.

This bidirectional interplay between mechanics and signalling has important implications for stem cell biology, tissue homeostasis and disease. In stem cell systems, mechanical competition may bias fate decisions toward differentiation, quiescence or senescence without overt apoptosis, providing a subtle mechanism for quality control. In cancer, the same mechanotransductive pathways may function either as tumour-suppressive surveillance mechanisms or be hijacked by super-competitor clones to eliminate surrounding healthy tissue. A major challenge for the field remains defining how mechanical thresholds, temporal dynamics and microenvironmental context determine whether Hippo–YAP-mediated competition promotes tissue integrity or pathological progression. Addressing these questions will be essential for translating cell competition into therapeutic strategies in regenerative medicine and oncology.

## Future perspectives

A deeper understanding of the context-dependent nature of cell competition will be essential for advancing highly personalized therapies. By profiling the competitive landscape within an individual’s tumour or degenerating tissue, it may become possible to design interventions that selectively tip the balance in favour of healthy or therapeutically engineered cells. Emerging technologies such as single-cell omics, spatial transcriptomics and live imaging will be instrumental in dissecting these interactions at high resolution and will drive the next generation of precision medicine strategies.

As the molecular mechanisms underlying cell competition are elucidated, new therapeutic targets and small-molecule modulators are likely to emerge. These could form the basis for drugs that enhance the competitive fitness of healthy cells or suppress that of diseased or malignant cells, offering a traditional pharmacological means to modulate cellular selection in vivo.

In regenerative medicine, one promising direction is the engineering of stem cells or other therapeutic cell types to act as super-competitors, enabling them to outcompete and replace dysfunctional cells. In oncology, similar principles may be applied by modifying immune cells, such as CAR T cells or CAR macrophages, to dominate and eliminate cancer cells within the tumour microenvironment. Enhancing the competitive potential of these engineered cells could significantly improve outcomes in both tissue regeneration and cancer therapy.

However, modulation of cell competition is unlikely to function as a standalone therapy. Its greatest clinical potential may lie in combination with established treatments, such as chemotherapy, radiotherapy or immunotherapy, to enhance therapeutic efficacy, reduce resistance or minimize collateral tissue damage. For example, chemotherapy may create selective pressures that favour the emergence of cell competition dynamics, which can then be therapeutically exploited.

In summary, the future of cell competition in therapeutics and regenerative medicine hinges on overcoming its inherent complexity through advanced molecular profiling, targeted drug development and cell engineering. If successful, this approach could mark a paradigm shift harnessing the body’s intrinsic capacity for cellular selection to maintain tissue health, suppress disease and promote regeneration.

## Abbreviations

BDNF: brain-derived neurotrophic factor
CNTF: ciliary neurotrophic factor
HSPCs: haematopoietic stem and progenitor cells
IL: interleukin
LATS1/2: large tumour suppressor kinases 1/2
MSCs: mesenchymal stromal cells
NGF: nerve growth factor
SCF: stem cell factor
TEAD: TEA domain transcription factors
YAP: yes-associated-protein
hPSCs: human pluripotent stem cells
